# Calcium channel blocker and risk of postoperation lymphatic-related complications among gynecologic malignances

**DOI:** 10.3389/fsurg.2022.939034

**Published:** 2023-01-05

**Authors:** Haote Jiang, Mengxiao Sun, Rongrong Shao, Shuyue Su, Yuyang Zhang

**Affiliations:** ^1^ The First School of Medicine, School of Information and Engineering, Wenzhou Medical University, Wenzhou, China; ^2^ Department of Gynecology, The First Affiliated Hospital of Wenzhou Medical University, Wenzhou, China

**Keywords:** CCB, calcium channel blocker, lymphadenectomy, lymph cyst, time of drainage, cervical cancer, endometrial carcinoma

## Abstract

**Purpose:**

This study was performed to assess the association of calcium channel blockers (CCB) and other potential factors with postoperative lymphatic-related morbidity in patients with cervical cancer and endometrial carcinoma.

**Methods:**

Patients diagnosed with cervical cancer or endometrial carcinoma with pelvic lymphadenectomy between January 2017 and January 2022 were enrolled. Postoperative lymphatic-related morbidity was evaluated by calculating the lymph cyst occurrence within 3 months after surgery and the duration of pelvic drainage. Univariate analyses evaluating the risk factors for lymphatic-related morbidity were performed.

**Results:**

Of a total of 251 patients, 52 patients were diagnosed with lymphatic cysts by B-ultrasound or computed tomography, and the duration of drainage from 110 patients exceeded the average number of days. Univariable analysis revealed that hypertension, CCB, and lymph node metastasis were independent predictors of postoperative complications.

**Conclusions:**

This study demonstrated that CCB may be a factor associated with the incidence of postoperation lymph cysts, and CCB usage prolongs the duration of pelvic drainage.

## Introduction

Gynecologic cancers, including ovarian, endometrial, and cervical cancers, are major types of malignancies for women worldwide. The incidence of cervical cancer in China is the highest among female malignant tumors (1, 2). Systematic pelvic lymph node dissection (PLND) remains an imperative surgical procedure in the treatment of gynecologic cancer. PLND not only improves staging accuracy but may also improve the prognosis.

Spontaneous contractions of smooth muscle cells in the lymphatic vessel walls are essential to maintain tissue homeostasis by draining fluid from peripheral tissues into circulation. Decreases in lymphatic pumping may reduce the velocity of lymphatic reflux and instigate lymph stasis or lymphedema. Lymphedema was first widely studied as a postoperative complication of breast cancer because of its high incidence and irreversibility. Of the patients treated for breast cancer, 41% develop lymphedema (3). Risk factors in patients with arm lymphedema include BIM > 30, radiation, advanced stage cancer, more invasive surgery, certain chemotherapy, infection, and so on (4).

Underlying the contractions are spontaneous action potentials and associated calcium ion (Ca^2+^) transients. Various studies have confirmed that the peacemaking mechanism relies on spontaneous transient depolarizations (STDs), which result in the opening of Ca^2+^-activated chloride channels (5).

Lymphatic leakage that commonly developed following pelvic lymph node dissection remains to be defined (6). It can occur anywhere along the pathway of lymph, which commences in all four extremities and the abdominal and peritoneal cavities. In 1994, Geissler et al first proposed the concept of chylous ascites, which is a rare form of ascites that results from the leakage of lipid-rich lymph into the peritoneal cavity.

Lymphatic leakage could cause some clinical symptoms such as limb lymphedema, prolonged lymphatic cyst, infection, and fever. The patients’ quality of life significantly deteriorated so as their sleep quality.

With the ongoing development of both laparoscopic technology and equipment, laparoscopic minimally invasive surgery is widely used in gynecological operations. Nevertheless, some studies have previously reported that CO^2^ pressure and ultrasonic knife coagulation during laparoscopic surgery could lead to transient occlusion of injured lymphatic vessels (6, 7). This obstruction subsequently causes lymphatic leakage during the patient's recovery. Due to the obstruction of lymphatic circulation and the presence of cavities in the retroperitoneum, localized lymphoid cyst are easily formed in the pelvis after systematic lymphadenectomy. Therefore, placing a pelvic drainage tube could drain the accumulated lymphatic fluid, and surgeons could observe whether there is active pelvic bleeding.

Several studies have analyzed the risk factors for the development of postoperative lymphatic leakage. Reported major risk factors for lymphatic leakage include having a high body mass index (BMI), radiation therapy, and having had lymph nodes removed, with the risk increasing with the number of lymph nodes removed. However, some of these risk factors remain ambiguous (8, 9).

A recent study showed that a novel risk factor of upper extremity lymphedema was associated with the treatment of breast cancer. In 2019, Stolarz et al. studied the effect of calcium channel blockers (CCB) on the risk of postoperative lymphedema in patients with malignant breast cancers for the first time. This retrospective study revealed that CCB exposure was significantly associated with an increased risk of lymphedema (10).

Unfortunately, throughout an array of standard postoperative lymphatic leakage research studies, there is still lack of consensus of lymphatic leakage. The researchers consider that drainage tube removal is a major factor affecting the morbidity of the patients’ physiological and psychological recovery as well as overall length of hospital stay. In our study, lymphatic leakage is defined as follows: the continuous drainage color of the postoperative pelvic drainage tube may be light yellow or chylous fluid, and the drainage fluid within 3–5 days postoperation is in excess of 300 ml/d, and other causes have been excluded (including urinary fistula).

## Method

This study is a retrospective case–control study, and block randomization will be adopted for random grouping. The 1,195 patients diagnosed with either cervical cancer or endometrial cancer between January 1, 2017, and January 1, 2022, who were treated in our hospital with PLND including dissection of the internal iliac, external iliac, common iliac, and obturator lymph nodes on both sides, were reviewed retrospectively. A total of 217 patients were excluded because the scope of lymph node dissection was beyond the pelvic cavity. Among the 978 patients remaining, 127 had hypertension. A total of 127 patients with a nonhypertensive gynecologic malignant tumor were randomly selected as control group. Due to the absence of pelvic imaging materials within 3 months after operation, 3 patients were excluded and a total of 251 patients were included.

The patients with cervical malignant tumors and endometrial cancers were divided into groups with strict limitations on the scope of lymph node dissection. For all included patients, data regarding age, weight, height, BMI, diagnosis of cancer, preexisting chronic disease (hypertension and diabetes), number of lymph nodes removed, duration of drainage (defined as the period elapsed from the first day after surgery to the day the drainage tube was removed), postoperative drainage volume, use of different antihypertension drugs, and postoperative therapy were collected.

Patients were sorted into two groups according to whether lymphatic cyst was found by imaging 3 months after operation. Considering the postoperative hospitalization time, we also grouped the patients according to the median time of duration of pelvic drainage.

The statistical analyses were performed using the SPSS statistics version 22.0 (IBM, Armonk, NY, United States) software programs. Continuous variables were presented as the mean and SD (normally distributed variables) or median. Categorical variables were presented as numbers and percentages. Variables with a value of *P *< 0.05 in the univariate analyses were included in the subsequent multivariate forward logistic regression analysis. Stepwise forward logistic regressions were used in multivariate analysis. Propensity score matching (PSM) was performed to eliminate the different distributions of variates among individuals in the two groups. PSM was conducted based on the logic of the propensity score and one-to-one nearest neighbor matching. The caliper was 0.02. All tests were two-sided, and differences were considered to be statistically significant at *P *< 0.05.

## Result

### Patient characteristics

In total, 122 patients diagnosed with cervical malignant tumors and 129 patients with endometrial cancers were enrolled in the present randomized controlled trial. With different kinds of cancer, patients were assigned to the lymph cyst group and nonlymph cyst group separately. Patient's age, BMI, hypertension, antihypertension drugs, blood routine, and biochemical indexes were similar between the two cohorts (Table 1).

**Table 1:** Patient's population.

Characteristics	Overall (*n* = 251)
Demographics	
Age	57.37 ± 10.00
BMI	24.40 ± 3.44
Cervical cancer	122 (48.6%)
Endometrial carcinoma	129 (51.4%)
Hypertension	127 (50.6%)
Diabetes	44 (17.5%)
CCB	65 (25.9%)
ACEI	23 (9.2%)
β	15 (6.0%)
Radiation	61 (24.3%)
Lymph node positivity	35 (13.9%)
Number of lymph node clearance	24.55 ± 9.88
Laboratory results	
WBC	6.97 ± 1.95
Neutrophil	4.46 ± 1.70
Lymphocyte	1.94 (1.50–2.32)
Monocyte	0.40 (0.32–0.49)
AST	24.95 ± 10.76
ALT	22.64 ± 16.22
ALB	43.20 ± 3.85
Cr	59.14 ± 12.79
Hb	125.02 ± 22.11

BMI, body mass index; CCB, calcium channel blockers; WBC, white blood cell; AST, Aspartate transaminase; ALT, Alanine transaminase; ALB, Albumin; Hb: Hemoglobin; Cr: Creatinine.

**Figure 1:**
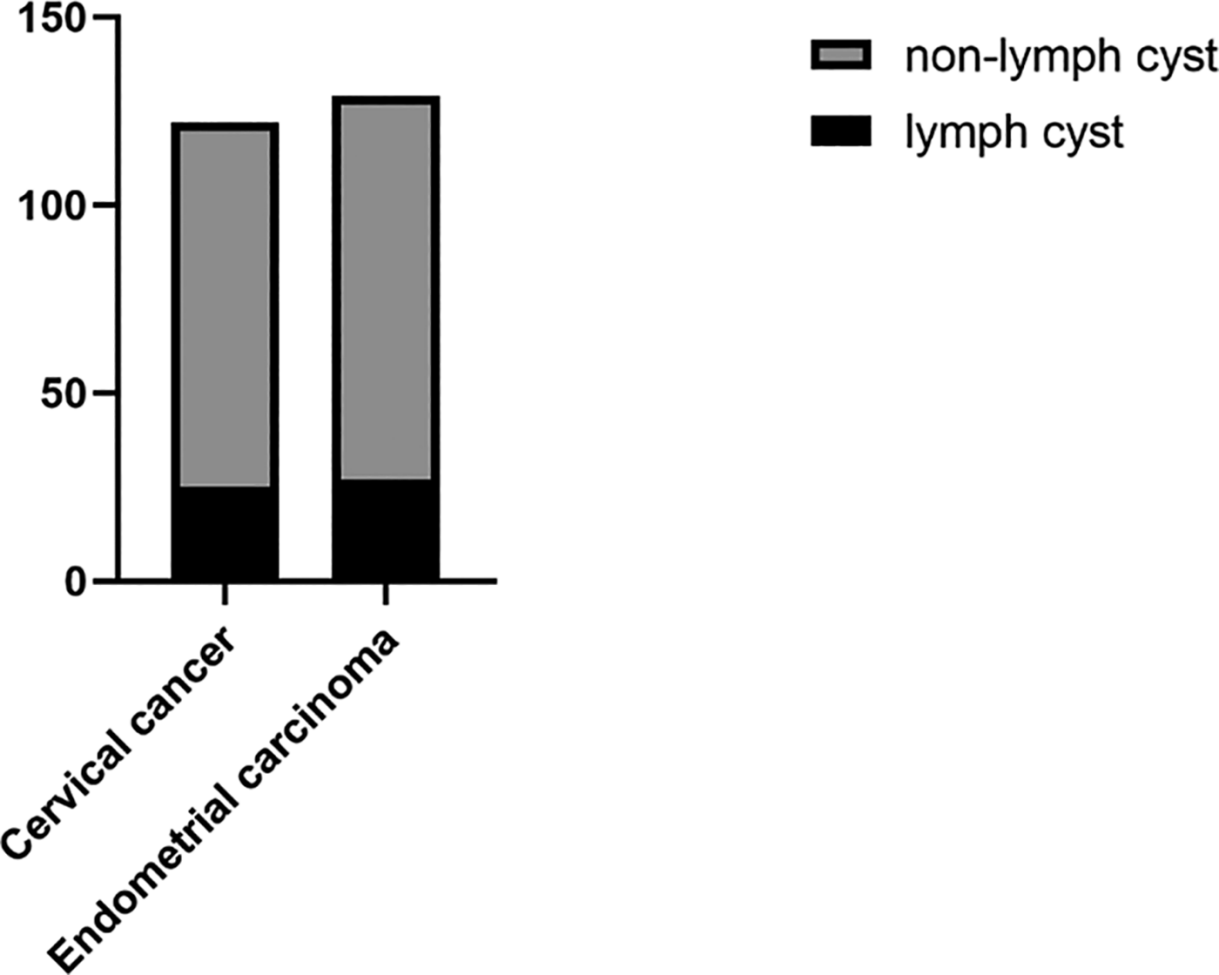
Incidence of lymph cyst.

The incidence of lymph cyst in cervical cancer was 19.7% and 21.9% in endometrial carcinoma. There was no significant difference between the two groups (Figure 1).

**Figure 2:**
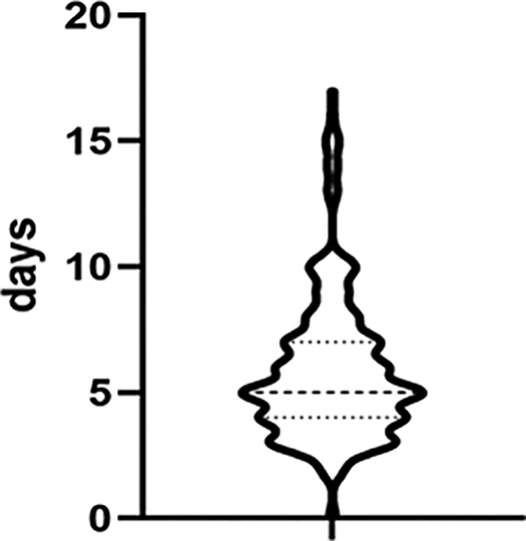
Duration of pelvic drainage..

The various lymph node dissections revealed 23.70 ± 8.5558 for cervical cancer and 25.36 ± 9.13 for endometrial malignance (*P* = 0.137). The characteristics of the two malignancies are outlined in Table 2. No difference was observed in BMI cervical cancer (CC): 24.01 ± 3.25 vs. endometrial carcinoma (EC): 24.77 ± 3.59; *P* = 0.079) and albumin (CC: 43.41 ± 4.36 vs. EC: 43.00 ± 3.29 s; *P* = 0.401) between the cervical cancer and endometrial cancer cohorts. Additionally, no significant difference was perceived regarding the duration of drainage. However, there was a statistically significant elevation in the rate of postoperation radiotherapy in the cervical cancer group (CC: 34.4% vs. EC: 14.7%, *P* = 0.04) (Table 2).

**Table 2:** Patients’ characteristics.

Characteristics	Cervical cancer (*n* = 122)	Endometrial carcinoma (*n* = 129)	*P*-value
Demographics			
Age	57.39 ± 11.65	57.35 ± 8.18	0.977
BMI	24.01 ± 3.25	24.77 ± 3.59	0.079
Medical history			
Hypertension	61 (50.0%)	66 (51.2%)	0.854
Diabetes	22 (18.0%)	22 (17.1%)	0.839
CCB	31 (25.4%)	34 (26.4%)	0.864
ACEI	13 (10.7%)	10 (7.8%)	0.425
β	8 (6.6%)	7 (5.4%)	0.706
Radiation	42 (34.4%)	19 (14.7%)	<0.001
Lymph node positivity	18 (17.1%)	17 (13.2%)	0.737
Number of lymph node removed	23.70 ± 8.55	25.36 ± 9.13	0.137
Laboratory results			
WBC	7.19 ± 2.19	6.77 ± 1.68	0.091
Neutrophil	4.62 ± 1.98	4.31 ± 1.37	0.160
Lymphocyte	1.98 (1.40–2.40)	1.82 (1.55–2.24)	0.290
Monocyte	0.41 (0.35–0.56)	0.38 (0.30–0.47)	0.470
AST	23.92 ± 10.21	25.92 ± 11.21	0.141
ALT	20.77 ± 12.73	24.40 ± 18.82	0.076
ALB	43.41 ± 4.36	43.00 ± 3.29	0.401
Cr	59.40 ± 14.56	58.90 ± 10.92	0.759
Hb	124.79 ± 23.62	125.24 ± 20.66	0.870

BMI, body mass index; CCB, calcium channel blockers; WBC, white blood cell; AST, Aspartate transaminase; ALT, Alanine transaminase; ALB, Albumin; Hb: Hemoglobin; Cr: Creatinine.

### Univariate and multivariate analysis for postoperative lymph cyst

The results of the univariate and multivariate analysis of factors associated with postoperative lymph cyst (within 3 months after surgery) are shown in Tables 3 and 4, respectively. In the univariate analyses for endometrial carcinoma, hypertension (OR = 2.779, *P* = 0.028), CCB (OR = 4.415, *P* = 0.001), ACEI (OR = 4.409, *P* = 0.028), and lymph node positivity (OR = 3.389, *P* = 0.027) were associated with the postoperative lymph cyst. Regarding cervical cancer, the result demonstrated that the number of lymph nodes removed (OR = 1.053, *P* = 0.049), neutrophil (OR = 1.293, *P* = 0.016), and NLR (ratio of neutrophils to lymphocytes) (OR = 1.428, *P* = 0.002) were linked with the development of a lymphatic cyst postoperation. Multivariate logistical regression models were created and shown that radiation (OR 2.593, 95% CI 1.014–6.629, *P* = 0.047) and neutrophil (OR 1.231, 95% CI 0.991–1.530, *P* = 0.060) were significantly associated with the postoperative lymph cyst in endometrial carcinoma, CCB (OR 4.915, 95% CI 1.860–12.984, *P* = 0.001) and radiation (OR 4.756, 95% CI 1.536–14.724, *P* = 0.007).

**Table 3:** Univiarate and multivariate analysis of cervical cancer for lymph cystis.

Characteristics	Univiarate		Analysis	Multivariate		Analysis
	OR	95% CI	*P*-value	OR	95% CI	*P*-value
Age	1.016	0.978–1.055	0.425			
BMI	0.970	0.844–1.115	0.667			
Hypertension	1.106	0.459–2.666	0.823			
Diabetes	1.176	0.387–3.573	0.774			
CCB	1.918	0.745–4.933	0.177			
ACEI	0.680	0.141–3.285	0.631			
β	1.319	0.250–6.967	0.745			
Radiation	3.136	1.271–7.742	0.013	2.593	1.014–6.629	0.047
Lymph node positivity	1.129	0.337–3.787	0.844			
Number of lymph node clearance	1.053	1.000–1.108	0.049			
WBC	1.205	0.995–1.460	0.057			
Neutrophil	1.293	1.048–1.595	0.016	1.231	0.991–1.530	0.060
Lymphocyte	0.537	0.277–1.041	0.066			
Monocyte	1.976	0.803–4.867	0.138			
AST	1.031	0.992–1.072	0.117			
ALT	1.012	0.980–1.045	0.474			
ALB	0.909	0.808–1.024	0.117			
Hb	0.984	0.968–1.001	0.065			

BMI, body mass index; CCB, calcium channel blockers; WBC, white blood cell; AST, Aspartate transaminase; ALT, Alanine transaminase; ALB, Albumin; Hb: Hemoglobin.

**Table 4:** Univiarate and multivariate analysis of endometrial carcinoma for lymph cystis.

Characteristics	Univiarate		Analysis	Multivariate		Analysis
	OR	95% CI	*P*-value	OR	95% CI	*P*-value
Age	1.041	0.988–1.096	0.130			
BMI	1.012	0.899–1.139	0.846			
Hypertension	2.779	1.115–6.927	0.028			
Diabetes	2.030	0.732–5.632	0.174			
CCB	4.415	1.796–10.853	0.001	4.915	1.860–12.984	0.001
ACEI	4.409	1.174–16.558	0.028			
β	0.001	0.001–	0.999			
Radiation	3.483	1.236–9.818	0.018	4.756	1.536–14.724	0.007
Lymph node positivity	3.389	1.146–10.025	0.027			
Number of lymph node clearance	1.046	0.998–1.096	0.061			
WBC	1.20	0.937–1.557	0.145			
Neutrophil	1.221	0.895–1.665	0.207			
Lymphocyte	1.381	0.700–2.724	0.351			
Monocyte	1.050	0.716–1.539	0.804			
AST	1.101	0.766–1.581	0.604			
ALT	1.007	0.972–1.044	0.684			
ALB	1.009	0.989–1.030	0.378			
Hb	0.975	0.856–1.110	0.698			

BMI, body mass index; CCB, calcium channel blockers; WBC, white blood cell; AST, Aspartate transaminase; ALT, Alanine transaminase; ALB, Albumin; Hb: Hemoglobin.

### Patient characteristics for duration of drainage

Hypertension, CCB, and lymph node metastasis were the risk factors according to the median duration of drainage (*P *< 0.001, *P* = 0.002, *P* = 0.035; Table 5).

**Table 5:** Patient characteristics for duration of drainage.

Characteristics	Duration of drainage ≤ 5 d (*n* = 141)	Duration of drainage > 5 d (*n* = 110)	*P*-value
Demographics			
Age	56.42 ± 9.22	58.58 ± 10.84	0.089
BMI	24.30 ± 3.28	24.54 ± 3.66	0.589
Medical history			
Hypertension	56 (39.7%)	71 (64.5%)	<0.001
Diabetes	28 (19.9%)	16 (14.5%)	0.272
CCB	26 (18.4%)	39 (35.5%)	0.002
ACEI	9 (6.4%)	1412.7%)	0.084
β	7 (5.0%)	8 (7.3%)	0.444
Radiation	39 (27.7%)	22 (20.0%)	0.160
Lymph node positivity	14 (9.9%)	21 (19.3%)	0.035
Number of lymph node clearance	24.82 ± 8.95	24.21 ± 8.81	0.588
Laboratory results			
WBC	6.91 ± 2.01	7.06 ± 1.88	0.555
Neutrophil	4.41 ± 1.82	4.52 ± 1.53	0.596
Lymphocyte	1.93 (1.46–2.32)	1.95 (1.55–2.31)	0.409
Monocyte	0.40 (0.32–0.50)	0.39 (0.31–0.48)	0.273
AST	24.58 ± 11.42	25.42 ± 9.89	0.542
ALT	22.23 ± 16.95	23.16 ± 15.29	0.651
ALB	42.98 ± 4.21	43.47 ± 3.32	0.320
Hb	124.59 ± 20.27	125.58 ± 24.34	0.726

BMI, body mass index; CCB, calcium channel blockers; WBC, white blood cell; AST, Aspartate transaminase; ALT, Alanine transaminase; ALB, Albumin; Hb: Hemoglobin.

### PSM

The PSM result between two groups is shown in Table 6. Seven variables (age, BMI, radiation, white blood cell, neutrophil, CCB, and the number of lymph node clearance) were selected to calculate the propensity score. After matching, only hypertension and the use of beta receptor blockers were associated with the occurrence of lymphoid cyst.

**Table 6:** Propensity score matching for cystis.

Characteristics	Before match			After match		
	Cystis group (*n* = 52)	Non-cystis group (*n* = 199)	*P*-value	Cystis group (*n* = 45)	Non-cystis group (*n* = 45)	*P*-value
Age	59.27 ± 10.91	56.87 ± 9.71	0.123	58.33 ± 11.06	62.53 ± 8.32	0.045
BMI	24.34 ± 3.77	24.42 ± 3.36	0.897	24.27 ± 4.00	24.55 ± 3.38	0.725
Hypertension	32 (61.5%)	95 (47.7%)	0.076	25 (55.6%)	38 (84.4%)	0.003
Diabetes	12 (23.1%)	32 (16.1%)	0.237	11 (24.4%)	8 (17.8%)	0.438
CCB	23 (44.2%)	42 (21.1%)	0.001	16 (35.6%)	18 (40.0%)	0.664
ACEI	7 (13.5%)	16 (8.0%)	0.228	6 (13.3%)	11 (24.4%)	0.178
β	2 (3.8%)	13 (6.5%)	0.467	1 (2.2%)	6 (13.3%)	0.049
Radiation	22 (42.3%)	39 (19.6%)	0.001	16 (35.6%)	16 (35.6%)	1
Lymph node positivity	11 (21.6%)	24 (12.1%)	0.081	9 (20.0%)	9 (20.0%)	1
Number of lymph node clearance	27.58 ± 9.08	23.76 ± 8.67	0.006	26.42 ± 7.80	24.27 ± 8.87	0.229
WBC	7.56 ± 2.30	6.83 ± 1.83	0.036	7.04 ± 1.81	7.49 ± 2.17	0.302
Neutrophil	5.05 ± 2.17	4.31 ± 1.52	0.024	4.53 ± 1.51	4.83 ± 1.93	0.413
Lymphocyte	1.80 (1.39–2.27)	1.97 (1.54–2.34)	0.554	1.77 (1.40–2.30)	1.97 (1.55–2.60)	0.366
Monocyte	0.44 (0.32–0.52)	1.80 (1.39–2.27)	0.296	0.44 (0.29–0.49)	0.39 (0.34–0.56)	0.491
AST	26.77 ± 13.52	24.47 ± 9.90	0.255	26.16 ± 13.41	23.22 ± 7.53	0.205
ALT	24.94 ± 17.54	22.04 ± 15.85	0.251	23.48 ± 15.71	19.58 ± 10.92	0.174
ALB	42.50 ± 4.30	43.38 ± 3.70	0.144	42.38 ± 4.36	42.60 ± 3.39	0.789
Cr	60.31 ± 12.57	58.84 ± 12.86	0.464	59.84 ± 12.34	62.78 ± 18.14	0.374
Hb	123.67 ± 24.98	125.37 ± 21.35	0.622	121.62 ± 25.75	126.98 ± 17.58	0.252

BMI, body mass index; CCB, calcium channel blockers; WBC, white blood cell; AST, Aspartate transaminase; ALT, Alanine transaminase; ALB, Albumin; Hb: Hemoglobin.

## Discussion

Calcium ion channels are present in the majority of cells of eukaryotic and prokaryotic organisms, which control a variety of cellular functions and regulate vital biological processes. Additionally, they also have been proposed to regulate a variety of biological and physiological processes, ranging from cellular secretions to electrical signaling. The discovery that nifedipine-sensitive voltage-dependent Ca^2+^ channels (VDCCs) control the regulation of the frequency of lymphatic contractions and the strength of contractions inspired researchers to attempt to develop an understanding of the relationship between lymphatic leakage and CCBs.

However, lymph node dissection was only an option for staging to particular patients, and it did not extend the survival time in patients with early-stage cancer. There is a heated debate in worldwide gynecological oncology circles about lymphadenectomy in early-stage epithelial ovarian cancer and endometrial cancer, with many beliefs in the necessity of a systematic lymphadenectomy for accurate staging. A number of research studies have reported that extensive lymph node dissection significantly increased postoperative respiratory complications and delayed patients’ rehabilitation.

In different studies, the rate of lymphedema complication in patients who underwent systematic pelvic lymphadenectomy for gynecologic malignancies was found to be 4%–35% (11, 12). A systematic pelvic and para-aortic lymphadenectomy still has been conventionally performed as a part of the whole procedure of surgery of gynecologic malignancies.

The existing postoperative complications related to lymphatic leakage after lymph node dissection were mainly studied in breast and gynecological cancers, and the main research was focused on the long-term complication—limb lymphedema caused by regional lymph node dissection. Our study was mainly focused on the short-term adverse reactions of patients after operation including postoperative time with drainage tube, length of hospital stay, and readmission rate within 30 days.

The mechanisms of lymphedema are complex and there is no consensus. In general, it requires the following structural condition for lymphatic leakage: the interruption of the lymphatic circulation pathway and the lymphatic pressure at the damaged site being greater than the tissue fluid pressure or intrabody pressure. Removal of the retroperitoneal lymph nodes during the operation of gynecologic malignancies may result in damage to some major lymphatic vessels and trunks with high pressure and a large flow, resulting in lymphatic leakage. The consensus regarding the most critical risk factors for lymphatic leakage is that the number of removed lymph nodes and having had adjuvant radiotherapy are the most strongly correlated to lymphatic leakage.

The larger the scope of lymph node dissection, the higher the probability of lymphatic vessel injury, which is a common cause of lymphatic vessel obstruction and dilatation. A dissection spanning a larger area is also correlated with the inadequate ligation of thick lymphatic vessels or injury of local lymphatic vessels during the operation.

However, different studies have reported variable outcomes from postoperative adjuvant therapy. Biglia et al. found that neither chemotherapy alone nor the association of consecutive chemotherapy and radiotherapy was associated with an increased incidence of lower limb lymphedema (13). In addition, there have been a few studies investigating the risk factors including BMI, nutritional status, number of removed lymph nodes, lymphedema, and adjuvant radiotherapy in patients having systematic lymphadenectomy (14, 15).

Due to the declining concentration of estrogen declining in most postmenopausal women, women with high blood pressure were mainly reported in the age group of 50 or older. These women were also prone to gynecology malignant tumors (16). CCBs are becoming one the most commonly prescribed drugs, which are gaining popularity due to their fewer side effects and acceptable tolerability. As studies into the role of calcium channel in the contractile function of the lymphatics carry on, association of CCB with the rate of postoperative upper limb lymphedema has been reported in some studies (10, 17). A common mechanism of action for antihypertensive medications is the regulation of calcium signaling and smooth muscle contraction, both of which are fundamental elements in normal lymphatic function. Different calcium channel blockers have been administered in the lymphatic vessels of mice to reduce the contraction frequency and weaken the contraction intensity (5).

At present, there is no effective treatment for lymphatic leakage. Treatment is predominantly divided into conservative treatment and surgical treatment. The main conservative treatments were prohibition, nutritional support, and somatostatin. The key to surgical treatment is to find the target lost lymphatic vessels and accurately ligate them. Due to the change of pelvic anatomy after the operation, the operation is very challenging. Some new methods were proposed. Some cases have reported embolization with fibrin glue and cyanoacrylate glue, but the effectiveness of these methods has not been confirmed. How to make these complications be anticipated as well as prevented in future is our immediate priority. Our study identified a new risk factor for postoperative complications in patients with pelvic lymph node dissection. The results of this study suggest that perhaps changing the use of perioperative antihypertensive drugs could affect the incidence of postoperative lymphoid cysts. However, a prospective cohort study with a larger sample size is needed to prove its reliability.

The major limitation of our study resides in several folds. First, it was a retrospective study, so a prospective study is needed to verify the accuracy of the prediction model. Second, the sample size was relatively small. There are differences in the incidence of lymphatic leakage among different types of cancer, and a larger sample size is needed for further study between groups. While some studies reported that hypertension has been associated with an increased risk of lymphedema, it remains unclear whether it is hypertension alone or hypertension treatment (17, 18). If the sample size is sufficient, it is necessary to analyze different antihypertensive drugs and rule out whether simple hypertension is an independent risk variable for postoperative lymphatic leakage-related complications.

## Data Availability

The raw data supporting the conclusions of this article will be made available by the authors, without undue reservation.
